# 

**Published:** 1823-09-01

**Authors:** 


### Contents

OF

No. 14, Vol. IV. for SEPTEMBER 1823.

Art. I.

Dr. Pring's Exposition of the Principles of Pathology and Therapeutics - - - - - - - - - - - - - - - - 241

II.

Mr. Swan's Enquiry into the Action of Mercury on the Living Body - - - - - - - - - - - - - - - - - 277

III.

Dr. O'Halloran on the Yellow Fever of the South and East Coasts of Spain - - - - - - - - - - - - - - 282

IV.

Analysis of the Article "Cas Rares," in the Dict. des Sciences Medicales - - - - - - - - - - - - - - - - 295

V.

DUBLIN HOSPITAL REPORTS.

(Concluded from p. 102.)

Art. 1. Dr. Crampton on the Application of Leeches to internal Surfaces - - - - - - - - - - - - - - 313
2. Dr. Byron on a Coagulum of Blood extracted from the Bladder - - - - - - - - - - - - - - - - - 316
3. Mr. Howship's Case of obstructed Œsophagus - - 317
4. Dr. Healy on a Paralytic Affection - - - - - - - 318
5. Case of perforation of the Perineum - - - - - *ib.*
6. Dr. Marsh on Jaundice - - - - - - - - - - - 319
7. Dr. Cuming's Case of diseased Heart - - - - - - 331
8. Dr. Cusack's Case of fatal Hæmorrhage - - - - - 334
9. Dr. O'Beirne on Traumatic Tetanus - - - - - - 335
10. Dr. Burgess on Bronchotomy - - - - - - - - - 339
11. Mr. Carmichael's Case of Hernia - - - - - - - 340
12. Dr. Scott's Case of dislocated Hip-joint - - - - - 342
13. Mr. Todd's Dissection of a Dislocation - - - - - 343
14. Mr. Todd on Diseases of the Lachrymal Gland - - 344
15. Dr. Henry Marsh on Diabetes - - - - - - - - 345

VI.

Dr. Barlow on the Physiology and Pathology of the Animal Frame - - - - - - - - - - - - - - - - - - - 350

VII.

Mr. Howship on Diseases of the Urinary Organs - - - - 369

VIII.

Dr. Paris and Mr. Fonblanque on Medical Jurisprudence - 383

IX.

Mr. Guthrie's Lectures on the Operative Surgery of the Eye 427

X.

QUARTERLY PERISCOPE.

1. Dr. Webster on Hooping Cough - - - - - - - - 449
2. Mr. Wardrop on Neuralgia - - - - - - - - - - 450
3. Mr. Sterry on strictured Colon - - - - - - - - - 451
4. Memoir on Œdema of the Glottis. By M. Bayle, M.D. - 452
5. Dr. Sauter's Case of extirpation of the Uterus - - - 463
6. Dr. Yeats's Case of painful Affection of the Brain - - 463
7. Dr. Bull's Case of Cancer of the Lip - - - - - - - 464
8. Baron Larrey's Memoir on Epilepsy - - - - - - - 465
9. Dr. Duchateau's Case of Abdominal Disease simulating Aneurism of the Heart and deceiving M. Corvisart - 468
10. Ulceration of the Cæcum communicating with an external Abscess, and destroying Life. *(Private correspond.)* 470
11. Mr. Cæsar Hawkins on Syphilitic Ulcers of the Larynx, observed at the Lock Hospital - - - - - - - 471
12. Ancient Facts better than Ancient Fictions - - - - - 479

XI.

Bibliographical Record - - - - - - - - - - - - - 481

XII.

INTELLIGENCE.

Associated Apothecaries and Surgeon-Apothecaries, &c. - - 483

XIII.

To Correspondents, &c. - - - - - - - - - - - - - 485

XIV.

Additional Subscribers - - - - - - - - - - - - - 486

